# Lipid and protein tumor markers for head and neck squamous cell carcinoma identified by imaging mass spectrometry

**DOI:** 10.18632/oncotarget.27649

**Published:** 2020-07-14

**Authors:** Janos Schmidt, Béla Kajtár, Kata Juhász, Mária Péter, Tamás Járai, András Burián, László Kereskai, Imre Gerlinger, Tamás Tornóczki, Gábor Balogh, László Vígh, Lászó Márk, Zsolt Balogi

**Affiliations:** ^1^ Institute of Biochemistry and Medical Chemistry, Medical School, University of Pécs, Pécs, Hungary; ^2^ Department of Pathology, Medical School, University of Pécs, Pécs, Hungary; ^3^ Institute of Biochemistry, Biological Research Center, Szeged, Hungary; ^4^ Department of Oto-Rhino-Laryngology, Medical School, University of Pécs, Pécs, Hungary; ^5^ MTA-PTE Human Reproduction Group, Medical School, University of Pécs, Pécs, Hungary; ^6^ Imaging Center for Life and Material Sciences, Medical School, University of Pécs, Pécs, Hungary

**Keywords:** Imaging mass spectrometry, tumor marker, lipid tumor marker, S100A8, S100A9

## Abstract

Head and neck squamous cell carcinoma (HNSCC) is the sixth most common cancer worldwide. To improve pre- and post-operative diagnosis and prognosis novel molecular markers are desirable. Here we used MALDI imaging mass spectrometry (IMS) and immunohistochemistry (IHC) to seek tumor specific expression of proteins and lipids in HNSCC samples. Among low molecular weight proteins visualized, S100A8 and S100A9 were found to be expressed in the regions of tumor tissue but not in the surrounding healthy stroma of a post-operative microdissected tissue. Marker potential of S100A8 and S100A9 was confirmed by immunohistochemistry of paraffin-embedded pathological samples. Imaging lipids showed a remarkable depletion of lysophosphatidylcholine species LPC[16:0], LPC[18:2] and, in parallel, accumulation of major glycerophospholipid species PE-P[36:4], PC[32:1], PC[34:1] in neoplastic areas. This was confirmed by shotgun lipidomics of dissected healthy and tumor tissue sections. A combination of the negative (LPC[16:0]) and positive (PC[32:1], PC[34:1]) markers was also applicable to uncover tumorous character of a pre-operative biopsy. Furthermore, marker potential of lysophospholipids was supported by elevated expression levels of the lysophospholipid degrading enzyme lysophospholipase A1 (LYPLA1) in the tumor regions of paraffin-embedded HNSCC samples. Finally, experimental evidence of 3D cell spheroid tests showed that LPC[16:0] facilitates HNSCC invasion, implying that HNSCC progression *in vivo* may be dependent on lysophospholipid supply. Altogether, a series of novel proteins and lipid species were identified by IMS and IHC screening, which may serve as potential molecular markers for tumor diagnosis, prognosis, and may pave the way to better understand HNSCC pathophyisiology.

## INTRODUCTION

Head and neck squamous cell carcinoma (HNSCC) includes a family of tumors arising from multiple locations (mouth, throat, larynx, sinuses and salivary glands) and is currently the sixth most common cancer worldwide. Tobacco and alcohol consumption are amongst high risk factors involved in HNSCC development. Meanwhile, human papillomavirus (HPV) or Epstein–Barr virus infections are associated with subgroups of HNSCCs. Despite complex treatment modalities (surgery, radiation, chemo-, photodynamic or targeted therapies) overall survival rates have only marginally increased for a long time. Improving treatment success rates would require earlier and more precise diagnosis as well as clinically applicable specific molecular markers of the tumor with prognostic value [1]. To date, molecular marker candidates of HNSCC include HPV, *EGFR* mutations, chemokine receptors, methylation markers, interleukins, which are mostly related to a subgroup of cases and also fail to provide a basis for specific and sensitive tumor identification [2]. Therefore, seeking molecular markers for early and precise diagnosis, reliable prediction of treatment results and recurrence rate remains a major goal in fighting HNSCC.

In light of inter- and intratumor heterogeneity that often causes treatment resistance and tumor relapse, application of multiregion sequencing and imaging approaches should become a regular practice [3]. For the same reasons, selection of marker signatures rather than single molecular targets has been suggested essential for precise diagnosis and prognosis [4]. A large dataset of omics results is now available for different tumor types, where serum, saliva, blood or solid samples have been fingerprinted for disease-associated molecular changes [5]. Tumors are characterized with a set of molecular changes of the proteome as well as the lipidome and metabolome [6]. Receptor mutations or changes in protein expression levels and localization have long been in the focus of tumor marker discovery, and recently lipidomic and metabolomic patterns have been outlining a molecular signature that may be of diagnostic and prognostic value [7–9]. However, ensemble analytical approaches are not capable of providing accurate localization-dependent information, hence it is still little known about the intratumoral and stromal localization of molecules. This is a major obstacle for understanding the molecular function and marker potential of critical proteins, lipids and metabolites in cancer. Imaging mass spectrometry (IMS) enables a label-free and sensitive detection and visualization of individual proteins and lipids in their fresh-frozen native tissue context. MALDI IMS specifically, provides images of 50–100 μm resolution for low molecular weight (LMW) proteins and lipids, where identity of each visualized molecule can be determined with the aid of omics or LC-MS techniques.

Screening for tumor-associated LMW protein and lipid changes in HNSCC tissue, here we identify S100A8, S100A9 and specific phospholipids to accumulate and lysophosphatidylcholine to be depleted in the tumor. Further we show that the lysophospholipid digesting LYPLA1 is accumulated in the tumor region of HNSCC tumors. Visualization of intratumoral heterogeneity points to the necessity of multiregion analysis and use of multiple molecular markers for reliable decisions.

## RESULTS

### S100A8 and S100A9 accumulation in the tumor tissue

**Figure 1:**
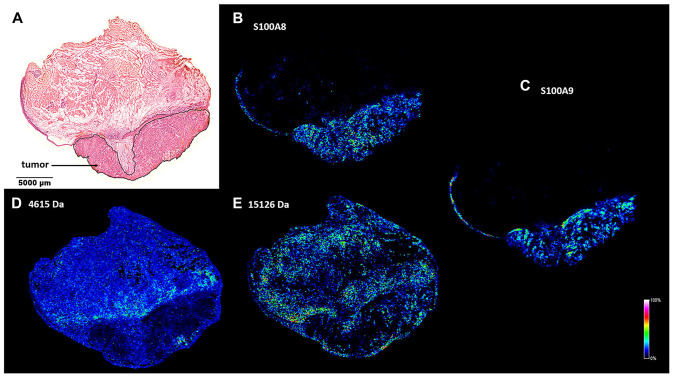
Spatial distribution of tumor marker candidate proteins in a HNSCC clinical specimen. (**A**) Pathological hematoxylin-eosin (H/E) staining of a fresh frozen tissue sample. Tumor region is marked. (**B** and **C**) MALDI MS image of S100A8 and S100A9 proteins in the healthy and tumor areas of the specimen. (**D** and **E**) MALDI MS image of unidentified protein examples of 4615 and 15126 Da.

A tumor mass causing destruction of the right side of the larynx had been detected in a 73-year-old patient, laryngoscopic biopsy revealed invasive keratinizing squamous cell carcinoma. Radiology showed localized tumor without lymph node involvement. Large enough to explore potential intratumoral heterogeneity, this HNSCC specimen was chosen for in-depth screening (Figure 1). 15 μm cryosections were prepared for pathological hematoxylin-eosin (H/E) staining and MALDI IMS (Figure 1), and the remaining tissue was kept frozen for further analysis. Tumor section of the specimen was marked based on morphological characterization of the H/E stained sample (Figure 1A). In an 80 μm resolution MALDI MS image series of the 4–17 kDa range of LMW proteins, S100A8 and S100A9 were essentially undetectable in the healthy stromal areas of the specimen, while they were present in the tumor and tumor stroma as well as in the healthy epithelial region (Figure 1A–1C). Immunoblotting and proteomics confirmed identity of the proteins (Supplementary Figure 1 and Supplementary Table 1). Additional unidentified protein examples at 4615 and 15126 Da were observed with a fairly negative expression in the tumor region, and heterogenous distribution throughout the stroma (Figure 1D and 1E).

**Figure 2:**
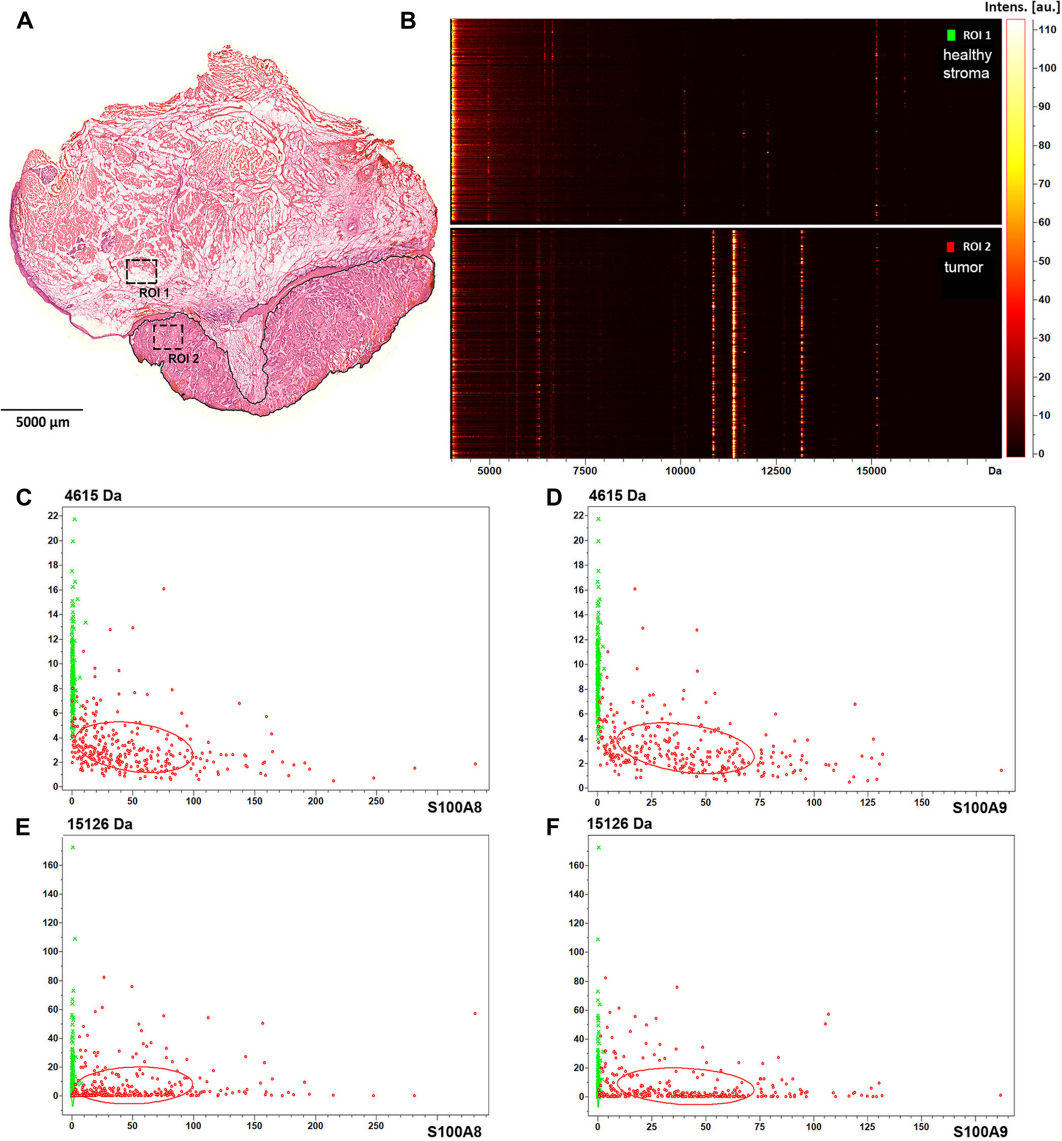
Protein expression and spatial distribution analysis of biopsy size regions of the tissue sample. (**A**) Healthy stroma and tumor region of interest are marked as ROI1 and ROI2 in the H/E stained section of the specimen, respectively. (**B**) Gel view format of image intensities of the detected proteins in the 4–17 kDa MW range. (**C**) Dual target intensities of pixels in the healthy (green) and tumor (red) ROIs are plotted for the 4615 Da protein and S100A8, (**D**) for the 4615 kDa protein and S100A9, (**E**) for the 15126 Da protein and S100A8, and (**F**) for the 15126 Da protein and S100A9.

Images of the above screening hits were further tested by analysing randomly selected biopsy size regions of the tissue sample (Figure 2A, ROIs). Image intensities, that are proportional to the expression levels of the detected proteins, were displayed in a gel view format. All image pixels of the healthy stroma ROI1 and the tumor ROI2 (Figure 2B) were shown as lines in the 4-17 kDa MW range. In support of visual inspection of the images (Figure 1), striking differences could be seen between the healthy stroma and tumor regions at 10829 Da and 13146 Da, which correspond to S100A8 and S100A9, respectively (Supplementary Figure 1 and Supplementary Table 1). Imaged at 11344 Da, the histone protein HIST2H4B was also accumulated in the tumor (Figure 2B). In Figure 2C–2F expression of S100A8 and S100A9 was probed against that of the proteins of 4615 Da and 15126 Da (Figure 1D and 1E) for both the healthy stroma (ROI1) and tumor (ROI2) regions of the specimen, respectively. Although displaying a heterogenous distribution within the examined ROIs, expression of either S100A8 or S100A9 clearly distinguished between healthy stroma and tumor areas, and proved to be a statistically reliable positive tumor marker of the specimen. Partially expressed also in the tumor, potential negative tumor markers with a MW of 4615 Da and 15126 Da (Figure 1D and 1E) had little added value to S100A8 and S100A9. Given some expression of S100A8 and S100A9 in the tumor stroma and the distant healthy epithelium, these regions proved not to be clearly distinguishable from the tumor (Supplementary Figures 2 and 3).

### HNSCC marker potential of S100A8 and S100A9

**Figure 3:**
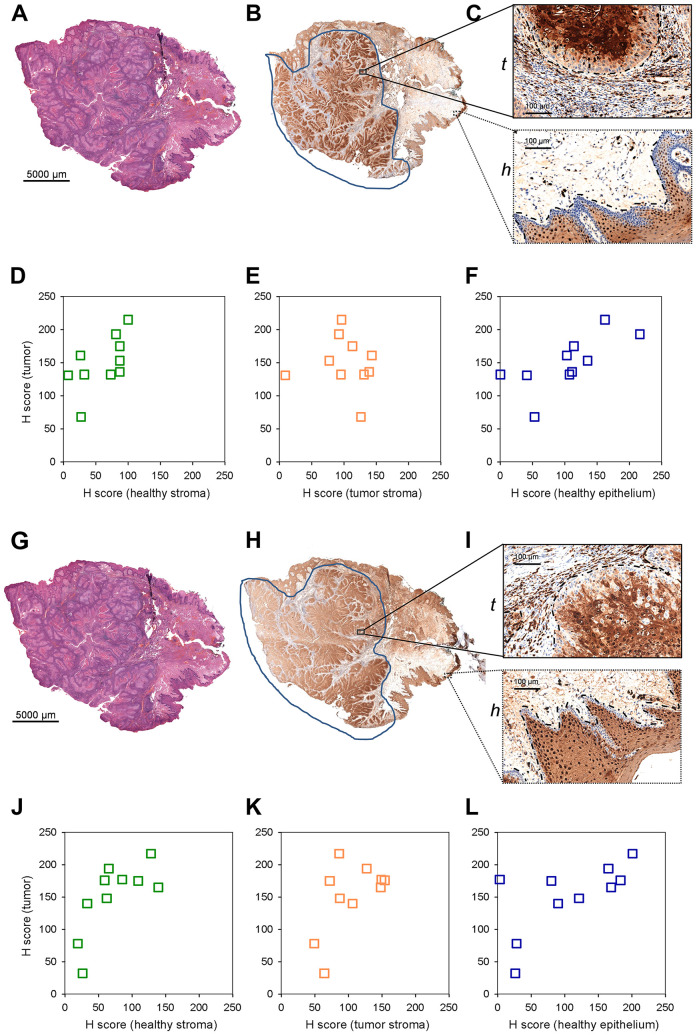
S100A8 and S100A9 staining of paraffin embedded clinical HNSCC samples by immunohistochemistry. (**A**) ^*^Hematoxylin-eosin staining of an example 4 mm tissue section. Note that this image is identical to (G). (**B**) Anti-S100A8 staining of the corresponding section. (**C**) Enlarged stained regions of the (t) tumor (tumor and tumor stroma) and (h) healthy (healthy epithelium and stroma) tissue areas. (**D**–**F**) Histopathological scoring of S100A8 staining in selected regions. Scored expression levels are displayed in (**D**) tumor vs. healthy stroma (**E**) tumor vs. tumor stroma and (**F**) tumor vs. healthy epithelium. (**G**) ^*^Hematoxylin-eosin staining of an example 4 mm tissue section. Note that this image is identical to Figure 3A, and shown for easier view only. (**H**) Anti-S100A9 staining of the corresponding section. (**I**) Enlarged stained regions of the (t) tumor (tumor and tumor stroma) and (h) healthy (healthy epithelium and stroma) tissue areas. (**J**–**L**) Histopathological scoring of S100A9 staining in selected regions. Scored expression levels are displayed in (J) tumor vs. healthy stroma (K) tumor vs. tumor stroma and (L) tumor vs. healthy epithelium.

S100A8 and S100A9 have been implicated in inflammation and tumor development, exerting a complex and multifactorial role [10]. To assess tumor marker potential of S100A8 and S100A9, a small cohort of paraffin-embedded pathological HNSCC samples (Supplementary Table 2) was tested by immunhistochemistry. 4 μm tissue sections were analysed by hematoxylin-eosin staining and probed with S100A8, S100A9 antibodies (Figure 3A, 3B, 3G, and 3H). Selected regions of the neoplastic and healthy tissue areas were subjected to histopathological scoring for determining S100A8 and S100A9 expressions in the tumor, tumor and healthy stroma as well as in the healthy epithelium (Figure 3C and 3I; and Supplementary Figures 4 and 5). Displaying expression levels in the tumor vs. in the healthy stroma revealed a clear accumulation of S100A8 and S100A9 in the tumor (Figure 3D and 3J), similar to that observed in the MS images (Figure 2C–2F). With a sizeable fraction of the examined samples showing clearly higher expression of S100A8 and S100A9 in the tumor vs. in the tumor stroma (Figure 3E and 3K) these tissue areas were statistically distinguishable (Supplementary Figure 6). Comparing the tumor to the healthy epithelium, the expression level of S100A8 and S100A9 in the tumor appeared slightly higher (Figure 3F and 3L), which was found statistically significant (Supplementary Figure 6). Altogether, imaging S100A8 or S100A9 expression allowed to differentiate the tumor tissue from the healthy stroma, and to a lesser extent distinguished between the tumor and the tumor stroma or the healthy epithelium.

### Specific lysophosphatidylcholine depletion and phospholipid accumulation in the tumor tissue

**Figure 4:**
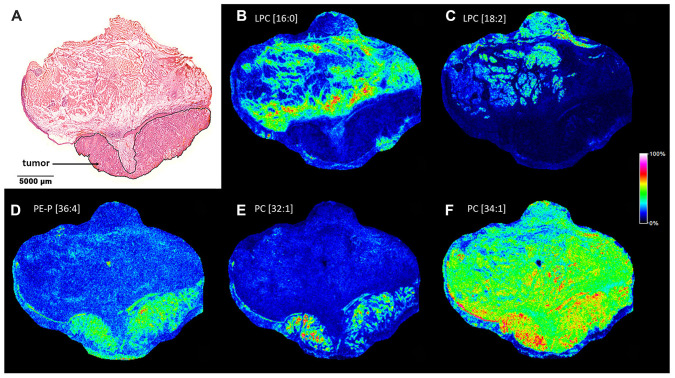
Lipid MS images of the HNSCC sample. (**A**) Pathological hematoxylin-eosin (H/E) staining of the fresh frozen tissue sample. Tumor region is marked. (**B**) MALDI MS image of palmitoyl-lysophosphatidylcholine LPC[16:0], (**C**) linoleoyl- lysophosphatidylcholine LPC[18:2], (**D**) plasmalogen phosphatidylethanolamine PE-P[36:4], (**E**) phosphatidylcholine PC[32:1], (**F**) PC[34:1]) of the section. Distribution and intensities of selected lipid species are visualized in the healthy and tumor tissue parts of the section.

Next, lipid MS images of the HNSCC sample were recorded, screening for tumor-specific lipid changes that may be associated with molecular changes with marker potential. As seen in Figure 4A–4C, palmitoyl- (LPC[16:0]) and linoleoyl- (LPC[18:2]) lysophosphatidylcholines were nearly completely absent from the tumor region of the specimen, while these lipid species were present in the healthy stroma in a discontinuous manner. On the contrary, some glycerophospholipid species such as plasmalogen phosphatidylethanolamine PE-P[36:4] and phosphatidylcholine PC[32:1] and PC[34:1] appeared to accumulate in the tumor area of the sample (Figure 4D–4F). Quantitative shotgun lipidomics was used to identify over 300 lipid molecular species from the healthy and tumor tissue parts. The corresponding lipid-ion adducts were queried and visualized by MALDI IMS. Although dissecting relatively large tissue parts for lipidomics did not enable to recapitulate high resolution information similar to that achieved by MS imaging, lipidomics and imaging data were in good agreement (Supplementary Figure 7). Furthermore, shotgun lipidomics revealed additional lipid species that were changed in the tumor as compared to its healthy tissue counterpart. Levels of lysophosphatidylcholine (LPC) and lysophosphatidylethanolamine (LPE) species were generally reduced in the tumor, resulting in decreased levels of the lipid classes LPC and LPE (7.08% to 1.06% and 5.50% to 1.64% for healthy vs. tumor, respectively). At the same time, an opposing trend was observed for phosphatidylethanolamine (PE) and to a lesser extent for phosphatidylcholine (PC) and plasmalogen phosphatidylethanolamine (PE-P) classes (9.41% to 18.72%, 30.22% to 35.19% and 9.85% to 11.74% for healthy vs. tumor, respectively). It should be noted that PE, PC and PE-P account for major constituents of the lipidome, therefore alteration even in a single lipid species may dramatically affect cellular physiology and tumor progression (see Figure 4F and Supplementary Figure 7 for PC[34:1]: 8.22% to 11.16% for healthy vs. tumor, respectively).

**Figure 5:**
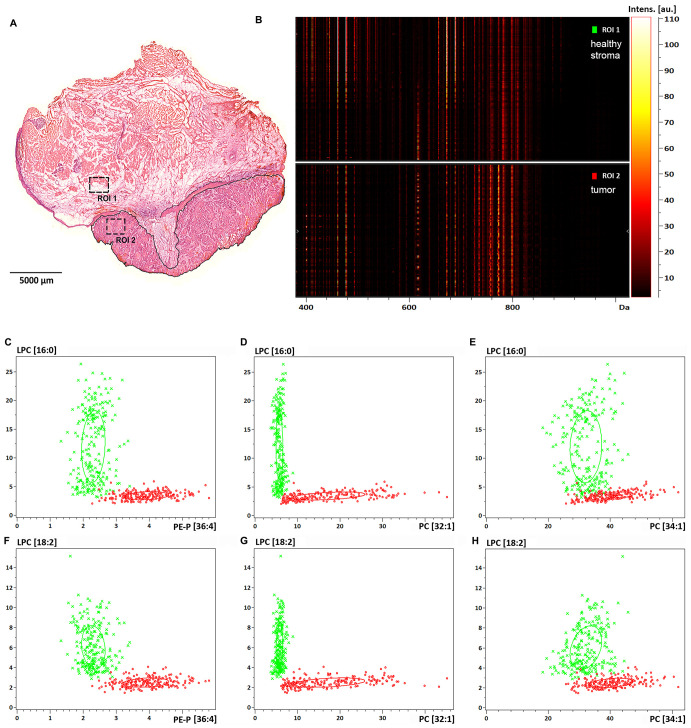
Lipid expression and spatial distribution analysis of biopsy size regions of the tissue sample. (**A**) Healthy stroma and tumor region of interest are marked as ROI1 and ROI2 in the H/E stained section of the specimen, respectively. (**B**) Gel view format of image intensities of the detected lipids in the 400-1000 Da MW range. (**C**) Dual target intensities of pixels in the healthy (green) and tumor (red) ROIs are plotted for LPC[16:0] and PE-P[36:4], (**D**) for LPC[16:0] and PC[32:1], (**E**) for LPC[16:0] and PC[34:1], (**F**) for LPC[18:2] and PE-P[36:4], (**G**) for LPC[18:2] and PC[32:1], (**H**) for LPC[18:2] and PC[34:1].

Further, distribution of the above lipid screening hits was statistically tested based on the MS images (Figure 4). Marking the same healthy stroma (ROI1) vs. tumor (ROI2) areas as for S100A8 and S100A9 analysis, lipid expression was displayed in a gel view format in the 400–1000 Da MW range (Figure 5A and 5B). Disappearance of lipid species at around 500 Da and a striking accumulation of more abundant lipid species in the 700-800 Da range were consistent with lysophospholipid depletion and phospholipid accumulation observed in the tumor (Figure 4 and Supplementary Figure 7). Probing the negative lipid marker candidates (LPC[16:0] or LPC[18:2]) against the positive ones (PE-P[36:4] or PC[32:1] or PC[34:1]), the healthy stroma vs. tumor tissues were clearly distinguished (Figure 5C–5H), indicating that these lipid species may be used in combination to assess more reliable pathological determination. Utilizing the same lipid markers, the tumor stroma vs. tumor tissues were statistically different as well (Supplementary Figure 8), however, the close values pointed to a tumor stroma adapting to the tumor tissue (compare Supplementary Figure 8 *vs*. Figure 5). Interestingly, the tumor region was little or not different from the healthy epithelium (Supplementary Figure 9).

**Figure 6:**
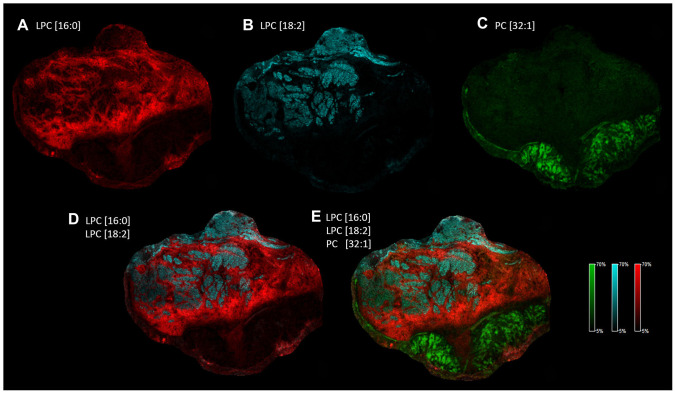
Heterogeneous distribution of specific LPC and PC species in the tissue sample. Distribution and intensity of specific lipid species visualized alone. (**A**) LPC[16:0] in red, (**B**) LPC[18:2] in blue, (**C**) PC[32:1] in green. Distribution and intensity of specific lipid species visualized in combination: (**D**) LPC[16:0] and LPC[18:2], (**E**) LPC[16:0], LPC[18:2] and PC[32:1].

A fairly heterogeneous, even discontinuous localization of the lipid marker candidates required thorough analysis of the spatial distribution of these lipid species (Figure 4). It was evident that in as small as 1 mm^2^ tissue ROIs (Figure 5A) there are low and high intensity pixels for lipids (Figure 5C–5E and 5F–5H), which point to a highly heterogeneous expression of specific lipid species (LPC[16:0], LPC[18:2]) throughout the tissue. To our surprise, LPC[16:0] and LPC[18:2] one after another appeared to mark different sections of the healthy stroma (Figures 5A, 6A, and 6B), which appeared to complement each other (Figure 6D). When used in combination, LPC[16:0], LPC[18:2] and PC[32:1] were seen as a remarkable set of negative and positive tumor markers of the post-operative tissue sample (Figure 6).

**Figure 7:**
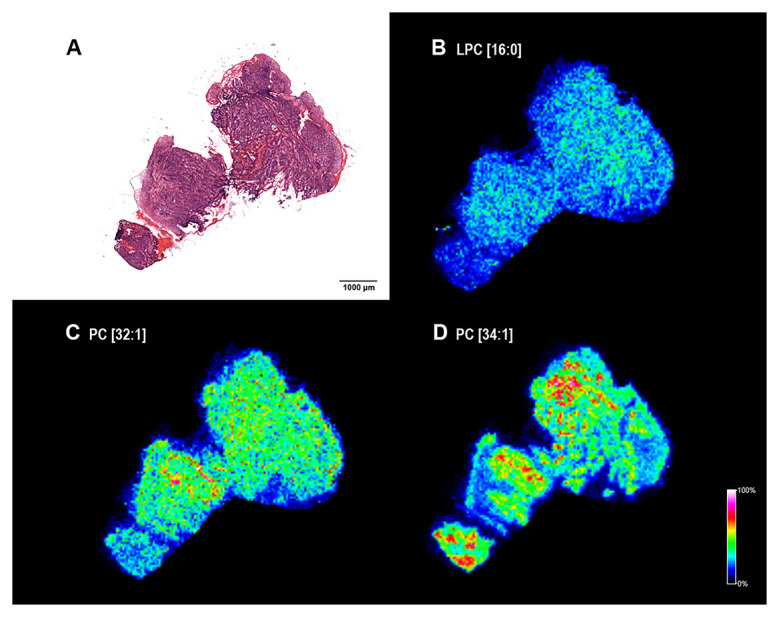
Lipid MS images of a HNSCC biopsy. (**A**) Hematoxylin-eosin (H/E) staining of the fresh frozen biopsy material. Note that only neoplastic tissue was identified. (**B**) MALDI MS image of palmitoyl-lysophosphatidylcholine LPC[16:0], (**C**) phosphatidylcholines PC[32:1] and (**D**) PC[34:1]) of the section. Distribution and intensities of selected lipid species are visualized in the tumor biopsy.

Next, we wondered lipid signatures of a freshly frozen fraction of a biopsy sample excised for routine pre-operative pathological characterization. Despite a relatively large sample taken from the hypopharynx of a 62-year-old patient no apparent healthy tissue part was revealed by H/E staining (Figure 7A). Cryosections were prepared for MALDI IMS and screening for lipids was performed as before. Among the screening hits of the large post-operative sample above (Figure 4) LPC[16:0], PC[32:1] and PC[34:1] were detectable in the biopsy, as well (Figure 7B–7D). As no healthy tissue region was recognized in the biopsy (Figure 7A) lipid expression levels of the tumor tissue could not be compared to expression levels in the healthy stroma (Figure 5A, 5D, and 5E). However, PC[32:1] and PC[34:1], that accumulate in the tumor (Figure 4E and 4F; and Supplementary Figure 7), could serve as positive expression references for LPC[16:0]. Similar to that seen in Figure 4, expressions of LPC[16:0] and PC[32:1], PC[34:1] were found low and high in the tumor tissue of the biopsy (Figure 7B–7D), respectively. Using this molecular set of negative and positive lipid markers, therefore, proved to be an efficient tool to identify the tumorous character of a pre-operative biopsy sample (Figures 6 and 7).

### HNSCC marker potential of the lysophospholipid lipase LYPLA1

**Figure 8:**
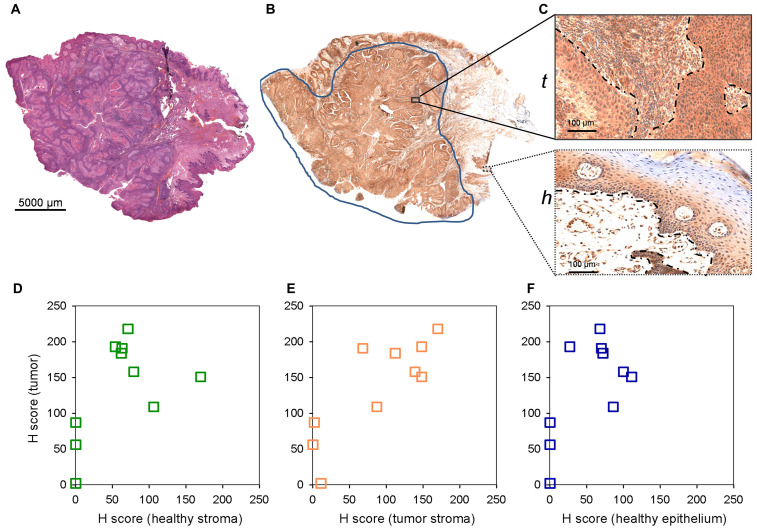
LYPLA1 staining of paraffin embedded clinical HNSCC samples by immunohistochemistry. (**A**) Hematoxylin-eosin staining of an example 4 mm tissue section. (**B**) Anti-LYPLA1 staining of the corresponding section. (**C**) Enlarged stained regions of the (t) tumor (tumor and tumor stroma) and (h) healthy (healthy epithelium and stroma) tissue areas. (**D**–**F**) Histopathological scoring of LYPLA1 staining in selected regions. Scored expression levels are displayed in (D) tumor vs. healthy stroma (E) tumor vs. tumor stroma and (F) tumor vs. healthy epithelium.

Pre-, or post-operative analysis of dissected tissue samples, or even *in situ* and real time lipid-based analysis during operation [11] have been emerging a real diagnostic possibility for clinicians. Nevertheless, immunohistochemistry of paraffin-embedded chemically crosslinked samples remains the routine of histopathology practice. Fixed samples are yet incompatible with lipid analysis, and there are no available antibodies recognizing the lysophospholipids LPC or LPE. Seeking an immunohistochemistry-compatible target we, therefore, assumed that lysophospholipid depletion in the tumor area of the HNSCC image may be associated with an increase in the expression of the lysophospholipid degrading enzyme lysophospholipid lipase A1 (LYPLA1). Using the same set of paraffin-embedded pathological HNSCC samples (Supplementary Table 2) that were tested for S100A8 and S100A9, expression and distribution of LYPLA1 was visualized by immunohistochemistry according to the scheme described earlier (Figures 3 and Figures 8A–8C). Selected regions of the neoplastic and healthy tissue areas were subjected to histopathological scoring for determining LYPLA1 expression in the tumor, tumor and healthy stroma as well as in the healthy epithelium (Figure 8D–8F and Supplementary Figure 10). Displaying expression levels in the tumor vs. in the healthy stroma revealed a clear accumulation of LYPLA1 in the tumor for most cases (Figure 8D and Supplementary Figure 11), largely consistent with MS image results for LPC[16:0] and LPC[18:2] (Figure 5C–5H). In addition, LYPLA1 expression was found higher in most tumor cases examined as compared to their tumor stroma counterparts (Figure 8E and Supplementary Figure 11), which was in good agreement with LPC[16:0] and LPC[18:2] distribution in the MS image (Supplementary Figure 8). Finally, LYPLA1 expression was found higher in most tumor cases examined as compared to their healthy epithelium counterparts as well (Figure 8F and Supplementary Figure 11). Altogether, expression and distribution of LPC[16:0], LPC[18:2] and LYPLA1 appeared to inversely correlate, supporting a lipid-based diagnosis of the tumor.

### Palmitoyl-lysophosphatidylcholine LPC[16:0] supports invasion and growth of HNSCC spheroids

**Figure 9:**
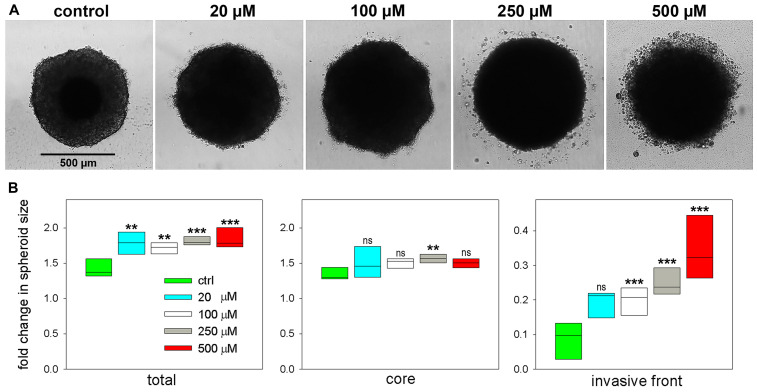
Effect of palmitoyl-lysophosphatidylcholine LPC[16:0] on tumor spheroid invasion and growth. 3D cell spheroids of hypopharynx squamous cell carcinoma origin were embedded in Matrigel matrix. Invasion and growth of spheroids are shown in the presence of increasing concentration of LPC[16:0] at 264 h. (**A**) Example images of spheroid cross-sections. (**B**) Size of spheroid cross-section areas are shown relative to values at 0 h (when embedding in Matrigel). Total spheroid size equals to the proliferative - necrotic core and the invasive zone. 95% CI is displayed, asterisks indicate statistical significance relative to control values.

Lysophospholipids have been proposed as potential fatty acid and lipid supply for promoting tumor development [12]. In order to better understand why the above lipid changes may happen, impact of LPC[16:0] administration on tumor progression was tested in a 3D cell spheroid experiment. Spheroids of FaDu cells of hypopharynx squamous cell carcinoma origin were embedded in Matrigel matrix at increasing concentration of LPC[16:0], then invasion and growth of spheroids were followed for 264 h. As seen in Figure 9A, without LPC in the surrounding Matrigel tissue invasion of a few single tumor cells was observed, which was, however, considerably increased by LPC[16:0]. Measuring spheroid cross-section areas relative to initial values revealed a significant increase in spheroid sizes at any LPC[16:0] concentrations tested (Figure 9B, total). Cores of the spheroids, i. e., the proliferative and necrotic zones without the invasive front, appeared to grow somewhat faster upon LPC treatment (Figure 9B, core; and Supplementary Figure 12). Noteworthy, after 168 h spheroids appeared to shrink to some extent, indicating starving, which was followed by a more pronounced invasion into the matrix (Supplementary Figure 12). Invasion of spheroids was clearly facilitated by LPC[16:0] in a concentration dependent manner (Figure 9B, invasion front).

## DISCUSSION

A limited number of available or candidate markers of HNSCC together with a high incidence rate of the disease urge to search for novel molecular tools for diagnosis or prognosis [2]. With mass spectrometry imaging-based screening here we identified a series of novel protein and lipid marker candidates for HNSCC. S100 proteins have been implicated in tumorigenic processes such as cell proliferation, metastasis, angiogenesis, and immune evasion… [10]. However, S100 family members of intracellular Ca^2+^ sensors exert multiple and complex functions, which may make their tumor marker potential uncertain. Each tumor subtype in fact, has its own S100 protein profile and signature, therefore must be evaluated on a specific basis. Mucosal epithelium, where squamous cell carcinoma originates from, is known to have high level of S100A8 and S100A9 at physiological conditions, which was reported to be present in the tumor as well. Interestingly, expression of S100A8 and S100A9 have been suggested to be reduced in HNSCC or oral cavity SCC specimens of patients with poor prognosis [13, 14]. Although healthy epithelial and tumor expressions were not different in our dataset, this may be dependent on locoregional tissue sampling, and in particular inflammatory status of the tumor microenvironment. This interpretation is supported by the fact that tumor and tumor stroma regions were not distinguishable either. However, S100A8 and S100A9 proved to be promising molecular markers differentiating between the healthy stroma and neoplastic regions.

An increasing body of evidence suggests that tumors depend on fatty acid and lipid supply. Bensaad *et al.* [15] has shown that hypoxia-induced fatty acid uptake and lipid droplet accumulation protects against ROS-generated lipotoxicity, hence confers a survival benefit and treatment resistance [16]. Fatty acid uptake mediated by CD36 has been found essential to initiate metastasis of human melanoma and breast cancer [17]. Moreover, most recently Vriens *et al.* [18] have revealed a fatty acid desaturation pathway that is independent of the classical stearoyl-CoA desaturase route, and converts palmitate to sapienate for supplying cancer cells with unsaturated fatty acids. Lysophospholipids have been found important fatty acid supplies for tumors *in vitro* [12]. In line with this, depletion of lysophospholipids, LPC in particular, from the blood and serum has become a diagnostic and prognostic value for various tumors [9, 19–28]. Moreover, lysophospholipids are important sources of the membrane forming phospholipids. Specifically, increased LPC acyltransferase (LPCAT1, LPCAT2) activities in neoplastic tissues have been proposed to confer tumor resistance through elevated PC production [29]. It should also be noted that lysophospholipids, if cleaved by autotaxin, yield lysophosphatidic acid (LPA) that exerts important tumor promoting functions through GPCR-mediated signaling [30]. Experimental evidence of this work demonstrates that lysophospholipids, specifically LPC[16:0] facilitates invasion and to some extent, growth of tumor spheroids of HNSCC origin. This is consistent with previous *in vitro* experimental data, reporting on a LYPLA1 dependent proliferation and migration of non-small cell lung cancer cells in 2D cultures [31]. Moreover, it is also in support of an earlier concept of a lysolipid dependent tumor progression examined in cell cultures of hypoxic and Ras-transformed cells [12].

Although a large body of experimental data support that LPC levels decrease and PC levels increase in different body fluids of tumor patients, very few studies have reported on intratumoral expression, and, in particular, localization of lipids. Ide *et al*. have shown accumulation of an abundant phospholipid species PC[36:1] in breast cancer tissue [32], which was not apparent for HNSCC in this study. Nevertheless, an increase in the major lipid species PC[34:1] has been reported for colorectal cancer [33], gastric cancer [34] and oral squamous cell carcinoma [35], and was revealed also for HNSCC of the hypopharynx by IMS here. Although further experimental data are required, it is tempting to speculate that relative enrichment of specific phospholipid species in the cancerous region may be a uniform feature of tumors which could be utilized as a molecular signature. Moreover, as compared to adjacent healthy areas, LPC[16:0] has been shown to be depleted from prostate cancer [36], gastric cancer [34] and HNSCC imaged in this study. Interestingly, Mirnezami *et al.* have reported an increase in LPC[16:0] and LPC[18:1] levels in colorectal cancer [33], and LPC levels were found unaltered in breast cancer earlier [32], indicating tumor type dependent depletion of LPC [37]. Reported for various tumors, depletion of LPC from blood and serum samples is, therefore, a likely consequence of an extensive lysolipid need for tumor progression (see also Figure 9). Accordingly, as body fluids appear to be indirect measures of lipid changes in the tumor it is also conceivable that LPC depletion could be earlier recognized in a biopsy sample of the suspected solid tumor. This approach is only applicable if biopsy collection is part of a diagnostic routine, such as examplified for HNSCC in this study. Further specific increase in PC[38:5] level in the tumor stroma of oral squamous cell carcinoma [35] was not detected in our images, however, this earlier finding is consistent with our results pointing to lipid reprogramming of the tumor stroma, including a partial accumulation of phospholipids. Moreover, a dramatic decrease in LPC and LPE levels in the tumor vs. healthy region of HNSCC was shown here, and a combination of lysophospholipid markers was applicable to differentiate between highly heterogenous healthy or tumor stroma and the tumor region. Interestingly, accumulation of LPC[16:0] was more pronounced in regions of the healthy stroma adjacent to the invasive front of the tumor (Figure 4), paralleled with an increase in LPA production measured in the healthy tissue sample (not shown). Although autotaxin has been shown not to have a direct tumor marker potential [38] it may therefore be a useful molecular component to mark the invading zone of the tumor.

Altogether, elevated expression of S100A8 and S100A9, upregulation of LYPLA1 and concomitant depletion of specific lysophospholipids, paralleled with an accumulation of specific phospholipids were identified as potential tumor markers via imaging mass spectrometry and immunohistochemistry-based screening of HNSCC specimens. Given that S100A8/9 support CD36-mediated fatty acid uptake [39] we propose that a set of molecular changes identified in this study may represent a fingerprint of fatty acid and lipid dependence of HNSCC pathophysiology. Together with serum and blood sample information, this molecular signature of HNSCC tumors might be used as future possible pre- or post-operative diagnostic and prognostic tools.

## MATERIALS AND METHODS

### Tissue samples and sectioning

Tumor samples were acquired after approval of the regional ethical committee (license No. 3382/2009) and informed consent of the patients. Fresh tumor tissues were stored at –80° C until processing. Freshly prepared 2% carboxymethyl cellulose embedding matrix was used for immobilization and a Leica CM1860 cryostat (Leica Microsystems GmbH, Wetzlar, Germany) was applied at –23° C for tissue sectioning. Tissues were cut at a thickness of 15 μm and thaw-mounted onto indium-tin-oxide-coated glass slides (Bruker Daltonics, Bremen, Germany). For protein identification, tissue sections were washed with ice-cold 70% and 90% aqueous ethanol solutions for 30 s, respectively, then dried under high purity stream of nitrogen gas.

### Matrix preparation

11 mg/ml of sinapinic acid (SA) was used for the protein identification, and 7 mg/ml of α-cyano-4-hydroxycinnamic acid (CHCA) matrix (SA; CHCA; Bruker Daltonics, Bremen, Germany) was used for the lipid identification processes. Matrices were dissolved in acetonitrile −0.2% aqueous trifluoroacetic acid solution (60/40, v/v) (Spectranal quality, Sigma-Aldrich, Budapest, Hungary). For matrix preparation, a self-developed, automated piezoelectric sprayer device was applied with 25 repetition cycle.

### Imaging mass spectrometry measurement

MS measurements were performed on an Autoflex Speed MALDI TOF/TOF mass spectrometer, which was equipped with a 1 kHz Smartbeam-II solid-state laser system (Bruker Daltonics, Bremen, Germany). Measurements were performed in positive linear mode using a detection range of m/z 3000–30000 in the case of the proteins, while in positive reflection mode using a detection range of m/z 380–3000 in the case of lipids. Tissues were measured at a lateral resolution of 80 μm, and 300 laser shots were summarized per each pixel. Data acquisition and evaluation processes were carried out by using FlexImaging 3.0 and FlexControl 3.4 software (Bruker Daltonics, Bremen, Germany).

### Protein identification

Microdissections of neoplastic and healthy tissues were used for the identification of proteins. Microdissection was performed manually under a Nikon SMZ 745T microscope (Nikon, Budapest, Hungary). Dissected tissue samples were homogenized and separated by one-dimensional SDS-polyacrylamide gel electrophoresis. The spots of interest were excised from the gel with a medical scalpel, and then transported into new Eppendorf tubes. After destaining, the proteins were alkylated, reduced and subjected to an overnight tryptic digestion. The resulting peptides were purified and concentrated with solid phase extraction, and then spotted onto a MALDI target plate. The peptide mass fingerprint-based proteomics identification was performed by an Autoflex II TOF/TOF mass spectrometer (Bruker Daltonics, Bremen, Germany).

### Lipid identification

Lipid standards were purchased from Avanti Polar Lipids (Avanti Polar Lipids Inc., AL, USA). Solvents for MS analysis and for tissue extraction were LC-MS grade and obtained from Sigma-Aldrich Co. 20 mg of tumor and non-neoplastic stroma tissues were homogenized in water using a bullet blender homogenizer (Bullet Blender Gold, Next Advance, Inc., Averill Park, NY, USA) in the presence of zirconium oxide beads (0.5 mm) at speed 8 for 5 min at 4° C. A portion of the homogenate (corresponding to 2 mg wet weight) was immediately subjected to a one-phase methanolic lipid extraction [40]. The homogenate was sonicated in 1 ml MeOH containing 0.001% butylated hydroxytoluene (as antioxidant) in a bath sonicator for 5 min, then shaken for 5 min and centrifuged at 10,000 g for 5 min. The supernatant was transferred into a new Eppendorf tube. The extracts were stored at –20° C. MS analysis was performed by an LTQ-Orbitrap Elite instrument (Thermo Fisher Scientific, Bremen, Germany) equipped with a robotic nano ion source TriVersa NanoMate (Advion BioSciences, Ithaca, NY, USA). Lipid classes and species were annotated using the lipid classification system [41] and individual lipids were identified by LipidXplorer software [42].

### Immunohistochemistry

4 μm sections were cut from formalin-fixed, paraffin-embedded biopsy samples. Immunohistochemical reactions were performed on dewaxed sections using a polymeric horseradish peroxidase-linker antibody conjugate system (Bond Polymer Refine Detection, Leica Biosystems). Following endogenous peroxidase blocking for 5 min and 20 min of antigen retrieval using a buffer of Tris-EDTA (Epitope Retrieval Solution 2, Leica Biosystems), sections were incubated with primary antibody for 15 min, followed by incubation with IgG linker and poly-HRP reagents for 8 min, respectively. Immunoreactions were revealed using a diaminobenzidine chromogen-hydrogen peroxide substrate for 10 min. Hematoxylin counterstain was applied for 5 min. All reagents were supplied by the manufacturer. S100A8 and S100A9 primary antibodies (R&D Systems, MAB4570 and MAB5578) were used in 1:2000 dilution, KO validated primary antibody against LYPLA1 (Abcam, EPR3667) was used in 1:800 dilutions. Slides were investigated by a histopathologist. Neoplastic and non-neoplastic epithelium and stroma were identified and scored for staining separately. H score was formulated for each compartment as follows: staining was scored as 0 (none), 1 (weak), 2 (moderate) or 3 (strong) for each cell investigated, the sum of 100 cells amounted to the H score. Data were analyzed by one-way Anova using Dunnett’s Multiple Comparison Test.

### Cell culture and 3D spheroid invasion experiments

Human hypopharynx squamous cell carcinoma cell line FaDu (ATCC HTB-43, American Type Culture Collection; Manassas, VI, USA) was cultured in DMEM (Biosera, Nuaille, France) supplemented with 10% fetal bovine serum (Biosera) at 37° C and 5% CO_2_. In *in vitro* 3D spheroid invasion experiments 5 × 10^3^ cells/well were concentrated at the U-bottoms of 96-well plates (Greiner Bio-One, Hungary) coated with poly-HEMA (6 mg/ml, Sigma-Aldrich, St. Louis, MI, USA). 48 h after seeding, 3D spheroids were formed, which were embedded in 3 mg/ml Matrigel Basement Membrane Matrix (Growth Factor Reduced w/o Phenol Red, Corning, NY, USA) containing 1% FBS. Brightfield images of spheroids were taken by a Nikon Eclipse inverted fluorescence microscope at 4× magnification at the indicated time points. Total image sizes of spheroid cross-sections were measured by Image J, while core sizes were determined by adaptive thresholding (MetaXpress, Molecular Devices). Size of the invasive front was calculated as a difference of total and core sizes. Spheroid size changes were expressed as relative to the values at 0 h (when embedding in Matrigel). Data were analyzed by one-way Anova using Dunnett’s Multiple Comparison Test.

### Immunoblotting

Healthy and tumor parts of fresh frozen samples were homogenized in RIPA buffer containing proteinase inhibitors, followed by protein concentration measurement using Bio-Rad Protein Assay Dye Reagent (Bio-Rad Laboratories Inc.). 50 μg protein was loaded onto reducing 15% SDS gel, followed by transfer onto PVDF membrane. Membranes were blocked with 5% nonfat dry milk in PBS-0.05% Tween-20 for overnight at 4° C. Immunoblotting was performed with primary antibodies anti-S100A8 and anti-S100A9 (R&D Systems) diluted in blocking solution incubating the membrane for 1 h at room temperature, followed by incubation with HRP conjugated anti-mouse secondary antibody (Sigma) for 1 h at room temperature. Signal was detected by Bio-Rad Clarity western ECL (Bio-Rad Laboratories Inc.) and autoradiography.

## SUPPLEMENTARY MATERIALS




