# Supplementary material for: Lipid and protein tumor markers for head and neck squamous cell carcinoma identified by imaging mass spectrometry
Source: Oncotarget. 2020 Jul 14;11(28):2702–17. doi: 10.18632/oncotarget.27649 (PMC7367650; doi:10.18632/oncotarget.27649)
Supplement: Supplementary file 1 [file oncotarget-11-2702-s001.pdf]

Lipid and protein tumor markers for head and neck squamous cell carcinoma identified by imaging mass spectrometry

SUPPLEMENTARY MATERIALS

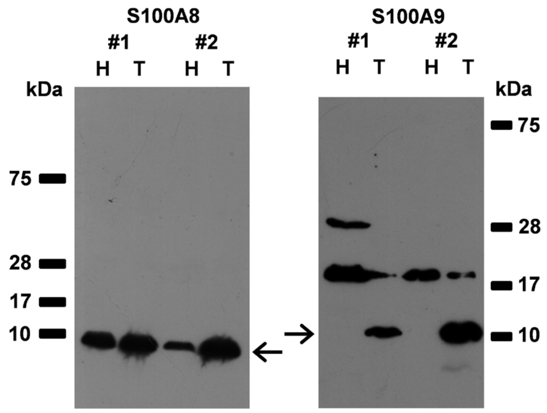

Supplementary Figure 1: S100A8 and S100A9 immunoblotting of healthy and neoplastic HNSCC tissue parts. Lysates of healthy (H) and tumor (T) parts of a fresh frozen clinical sample were probed with anti-s100A8 and anti-s100A9 antibodies by western blot. Arrows point to S100A8 and S100A9, respectively. Equal amounts of proteins were loaded.

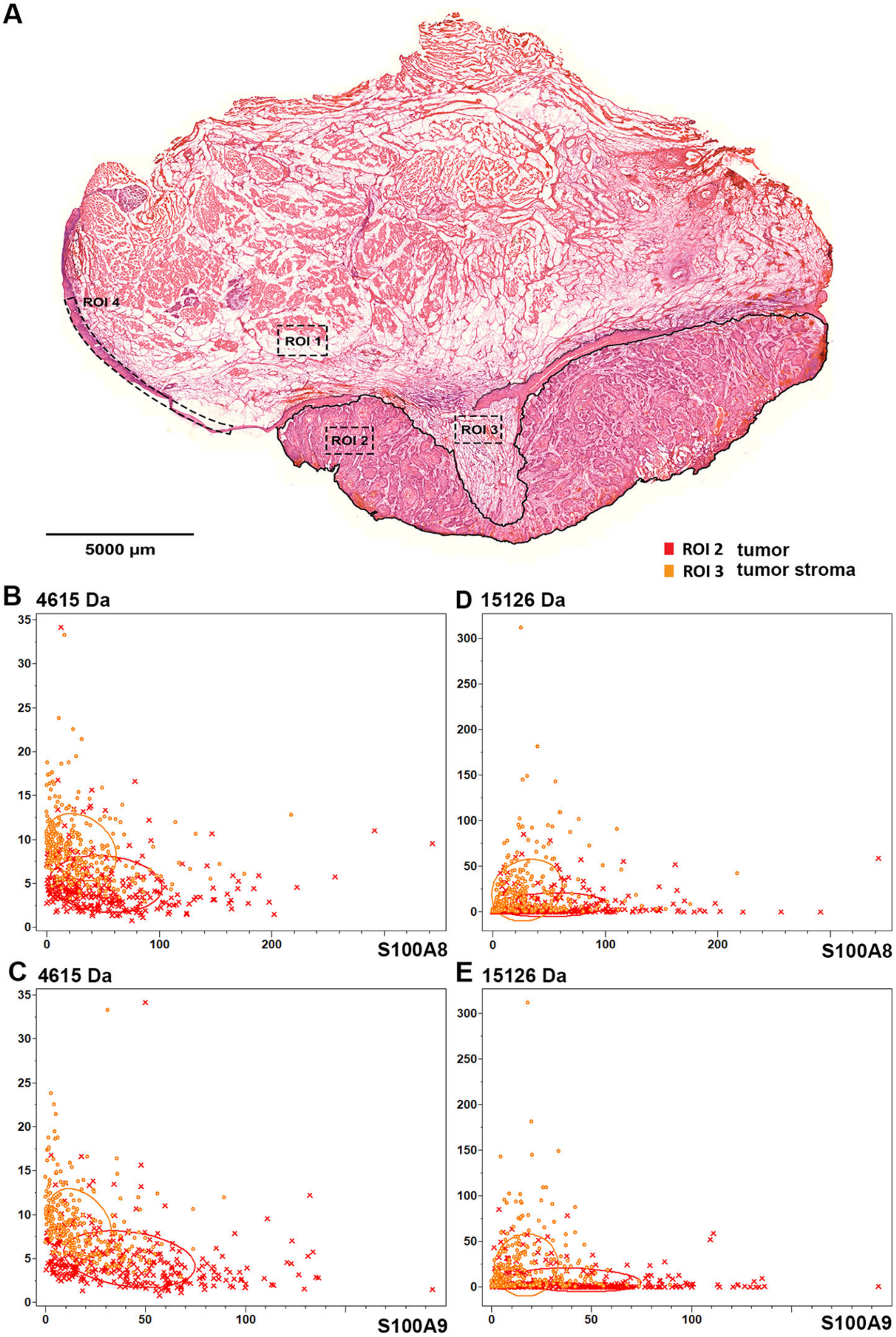

Supplementary Figure 2: Protein expression and spatial distribution analysis of biopsy size regions of the tissue sample. (**A**) Tumor stroma and tumor region of interest are marked as ROI3 and ROI2 in the H/E stained section of the specimen, respectively. (**B**) Dual target intensities of pixels in the tumor stroma (orange) and tumor (red) ROIs are plotted for the 4615 Da protein and S100A8, (**C**) for the 4615 kDa protein and S100A9, (**D**) for the 15126 Da protein and S100A8, and (**E**) for the 15126 Da protein and S100A9.

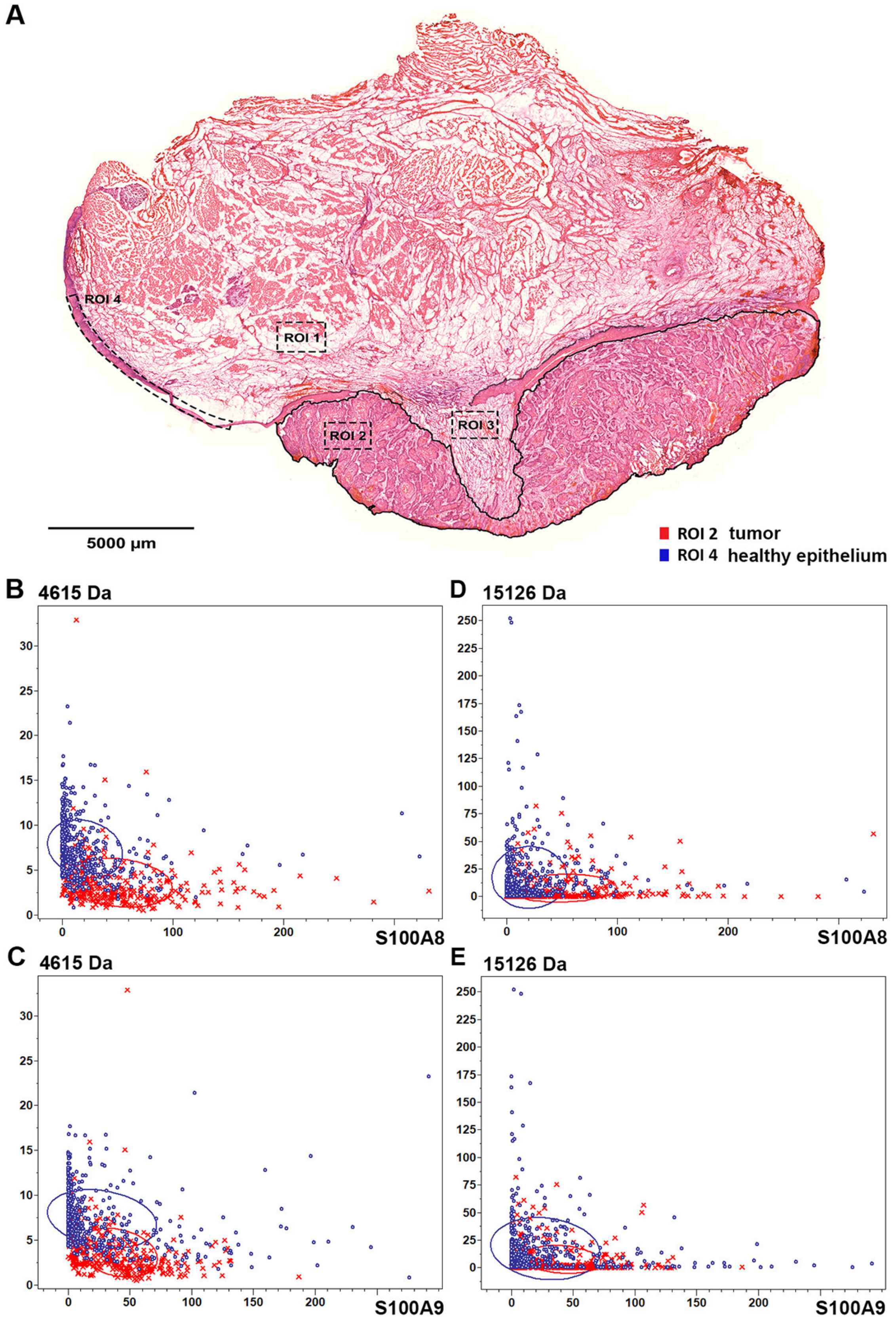

Supplementary Figure 3: Protein expression and spatial distribution analysis of biopsy size regions of the tissue sample. (**A**) Healthy epithelium and tumor region of interest are marked as ROI4 and ROI2 in the H/E stained section of the specimen, respectively. (**B**) Dual target intensities of pixels in the tumor stroma (orange) and tumor (red) ROIs are plotted for the 4615 Da protein and S100A8, (**C**) for the 4615 kDa protein and S100A9, (**D**) for the 15126 Da protein and S100A8, and (**E**) for the 15126 Da protein and S100A9.

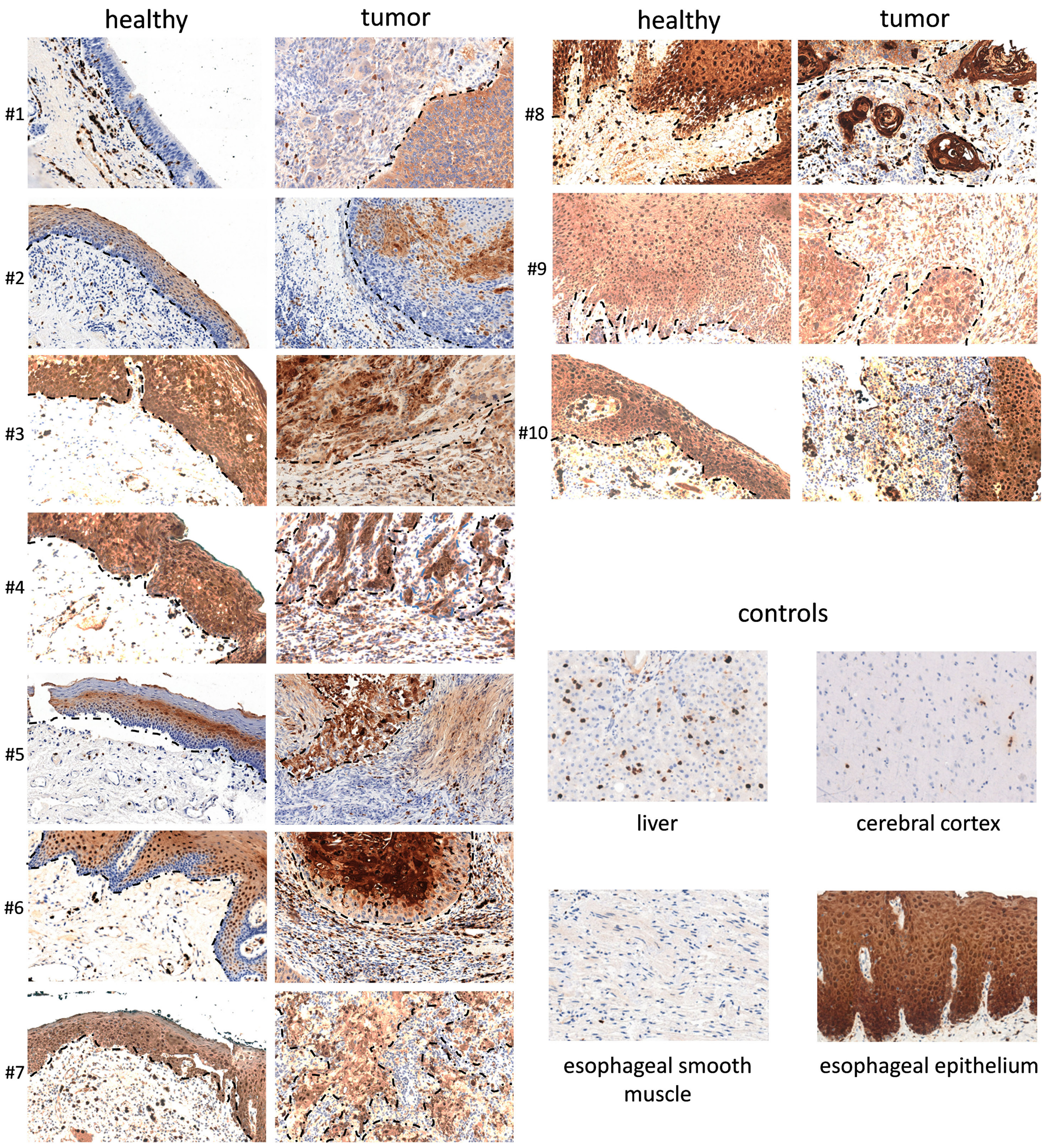

Supplementary Figure 4: S100A8 staining of paraffin embedded clinical HNSCC samples by immunohistochemistry. S100A8 staining of 4 µm tissue sections in healthy (healthy epithelium and stroma) and tumor (tumor and tumor stroma) regions of clinical samples. Negative (liver, cerebral cortex, esophagus muscle) and positive (esophagus epithelium) tissue control stainings are shown.

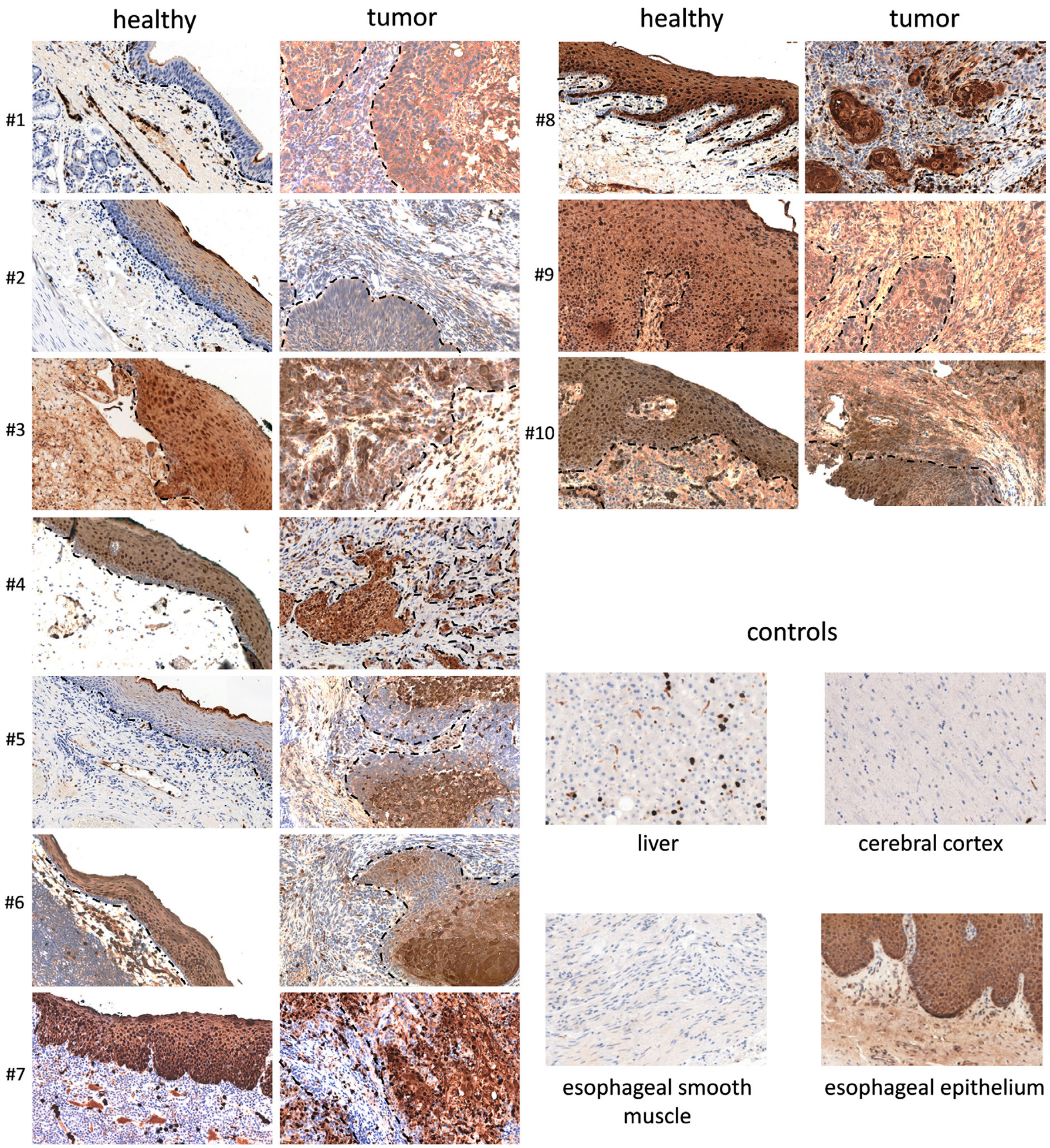

Supplementary Figure 5: S100A9 staining of paraffin embedded clinical HNSCC samples by immunohistochemistry. S100A9 staining of 4 µm tissue sections in healthy (healthy epithelium and stroma) and tumor (tumor and tumor stroma) regions of clinical samples. Negative (liver, cerebral cortex, esophagus muscle) and positive (esophagus epithelium) tissue control stainings are shown.

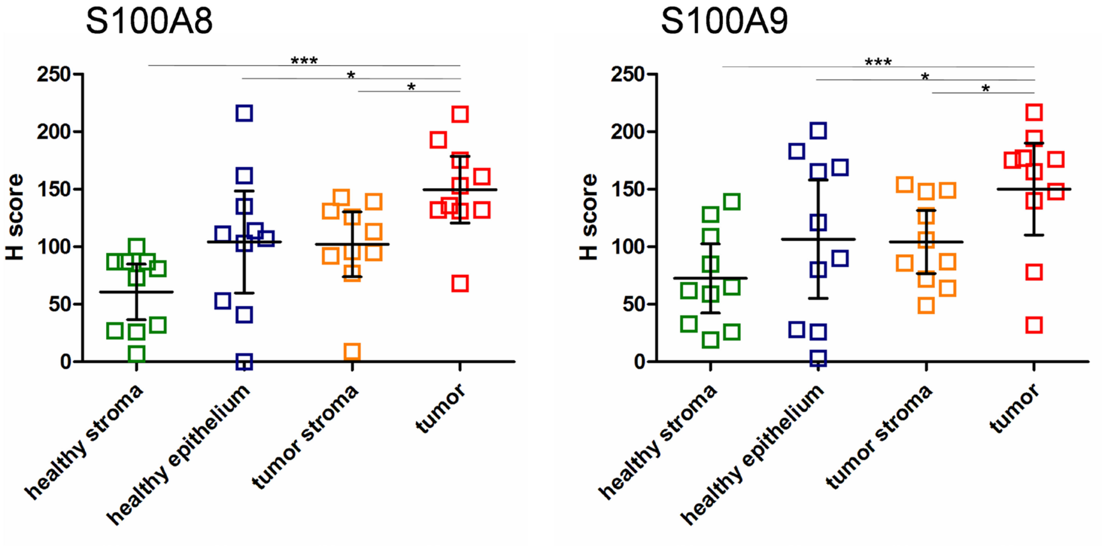

Supplementary Figure 6: H-scored expression levels of S100A8 and S100A9 staining in healthy and tumor regions of clinical samples.

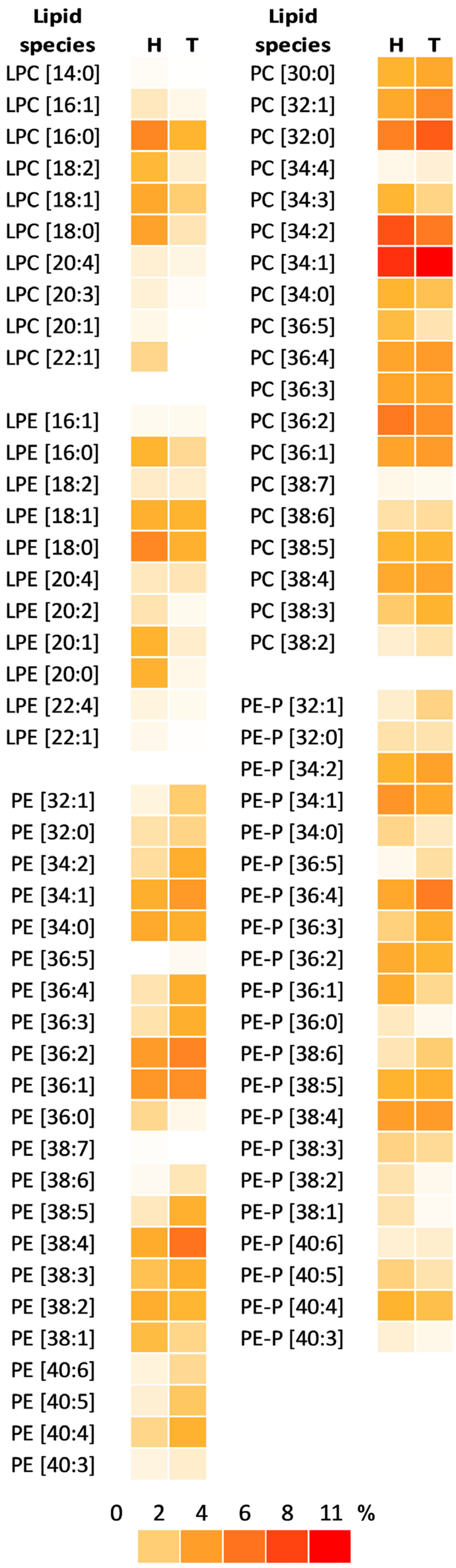

Supplementary Figure 7: Shotgun lipidomics of the healthy and tumor tissue counterparts. Mol % of LPC, LPE, PE, PC, and PE-P species are displayed in a heatmap format relative to total polar lipid content.

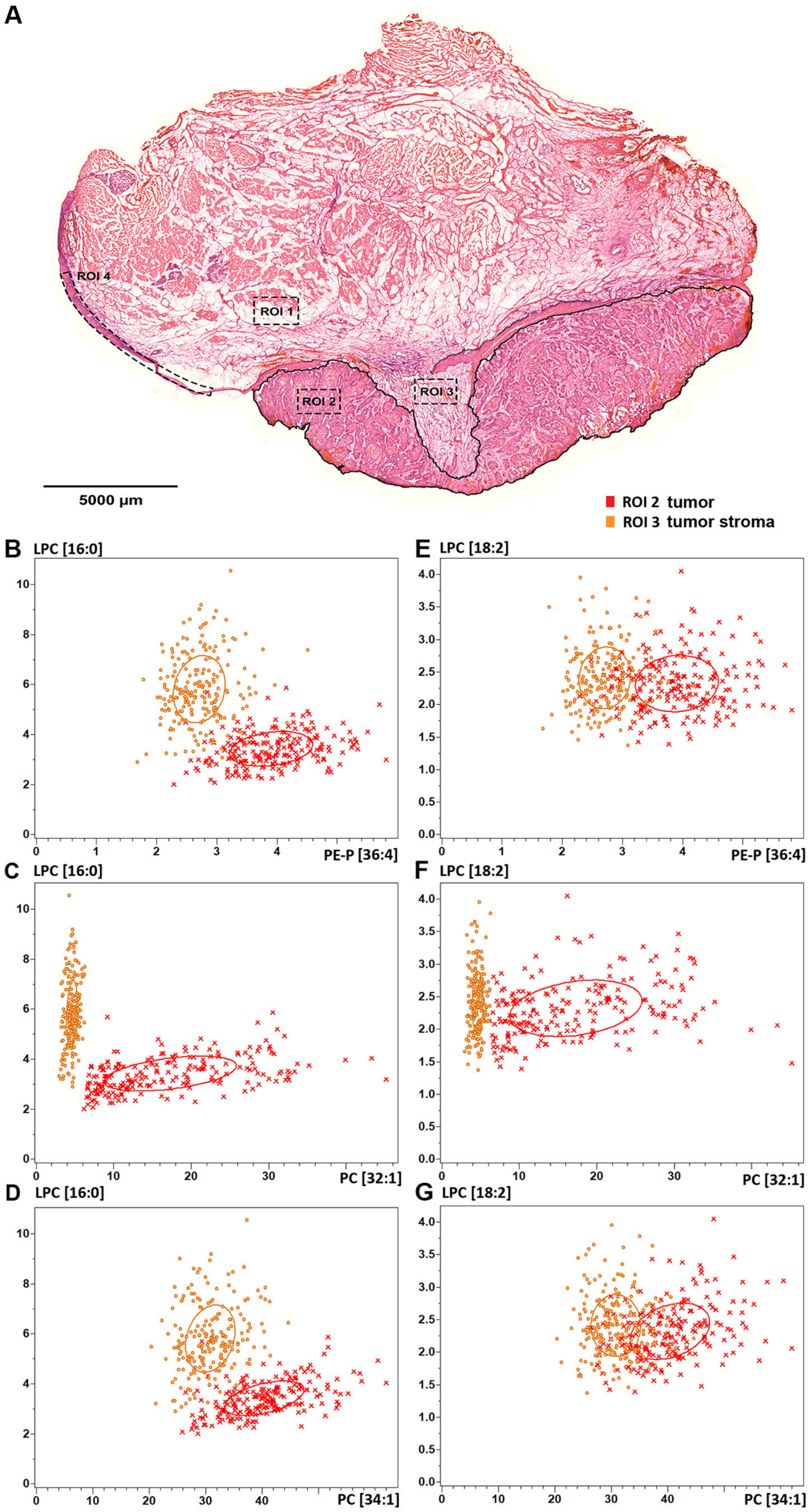

Supplementary Figure 8: Lipid expression and spatial distribution analysis of biopsy size regions of the tissue sample. (**A**) Tumor stroma and tumor region of interest are marked as ROI3 and ROI2 in the H/E stained section of the specimen, respectively. (**B**) Dual target intensities of pixels in the tumor stroma (orange) and tumor (red) ROIs are plotted for LPC[16:0] and PE-P[36:4], (**C**) for LPC[16:0] and PC[32:1], (**D**) for LPC[16:0] and PC[34:1], (**E**) for LPC[18:2] and PE-P[36:4], (**F**) for LPC[18:2] and PC[32:1], (**G**) for LPC[18:2] and PC[34:1].

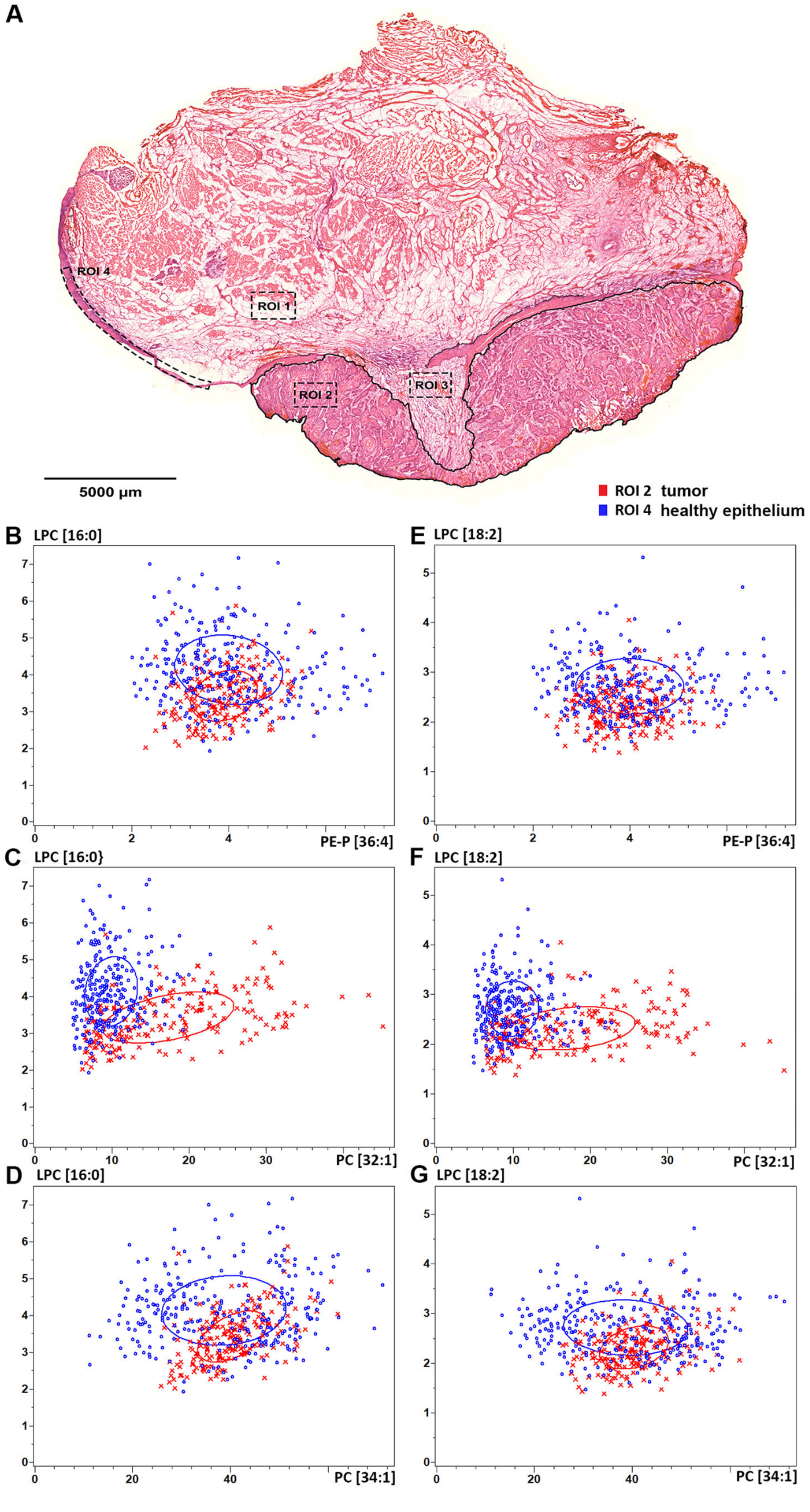

Supplementary Figure 9: Lipid expression and spatial distribution analysis of biopsy size regions of the tissue sample. (**A**) Healthy epithelium and tumor region of interest are marked as ROI4 and ROI2 in the H/E stained section of the specimen, respectively. (**B**) Dual target intensities of pixels in the healthy epithelium (blue) and tumor (red) ROIs are plotted for LPC[16:0] and PE-P[36:4], (**C**) for LPC[16:0] and PC[32:1], (**D**) for LPC[16:0] and PC[34:1], (**E**) for LPC[18:2] and PE-P[36:4], (**F**) for LPC[18:2] and PC[32:1], (**G**) for LPC[18:2] and PC[34:1].

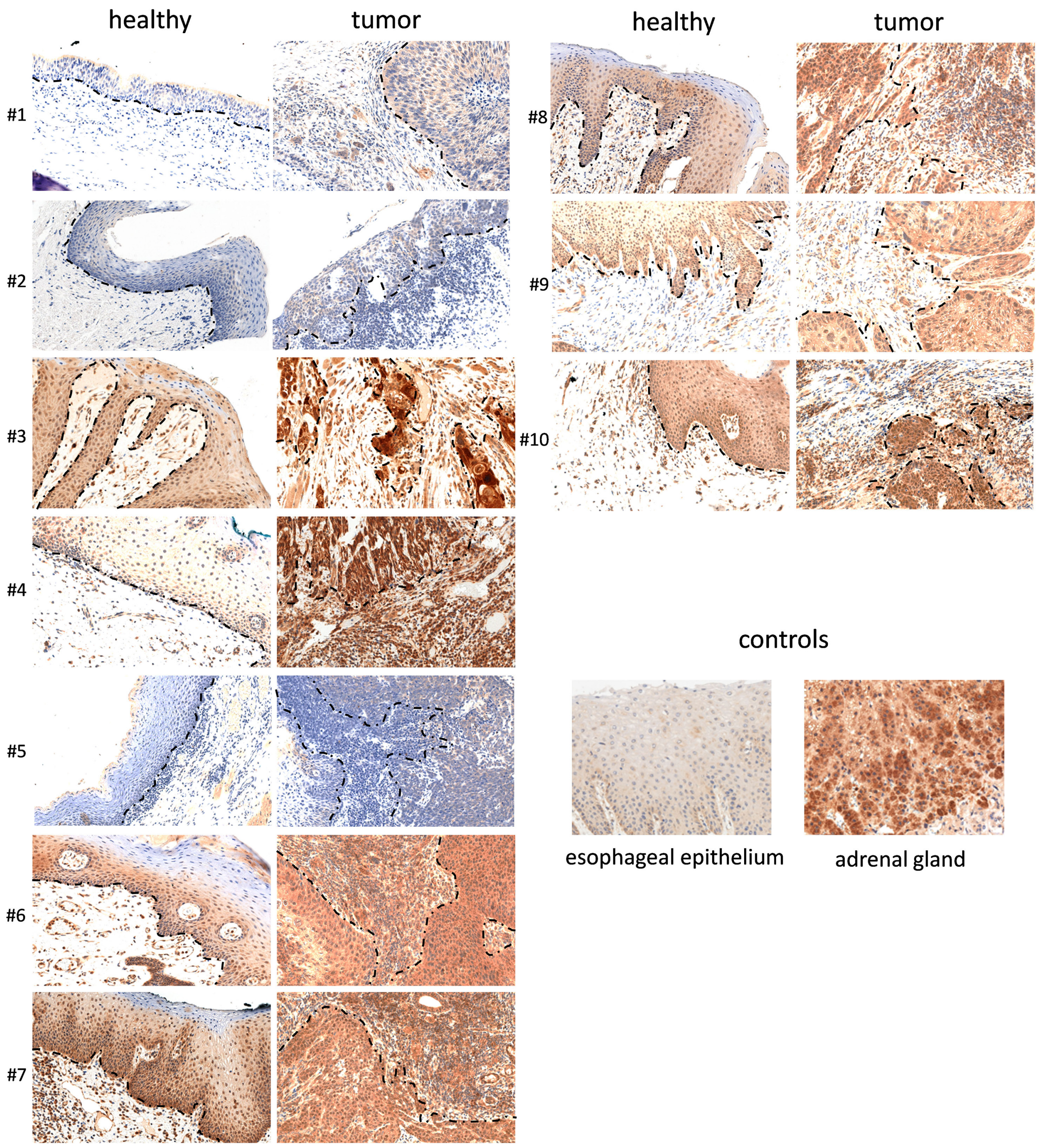

Supplementary Figure 10: LYPLA1 staining of paraffin embedded clinical HNSCC samples by immunohistochemistry. LYPLA1 staining of 4 mm tissue sections in healthy (healthy epithelium and stroma) and tumor (tumor and tumor stroma) regions of clinical samples. Negative (esophagus) and positive (adrenal gland) tissue control stainings are shown.

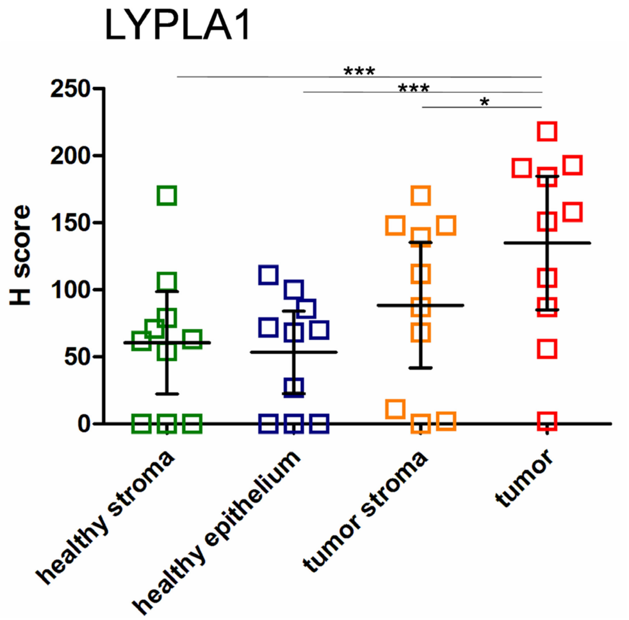

Supplementary Figure 11: H-scored expression levels of LYPLA1 staining in healthy and tumor regions of clinical samples.

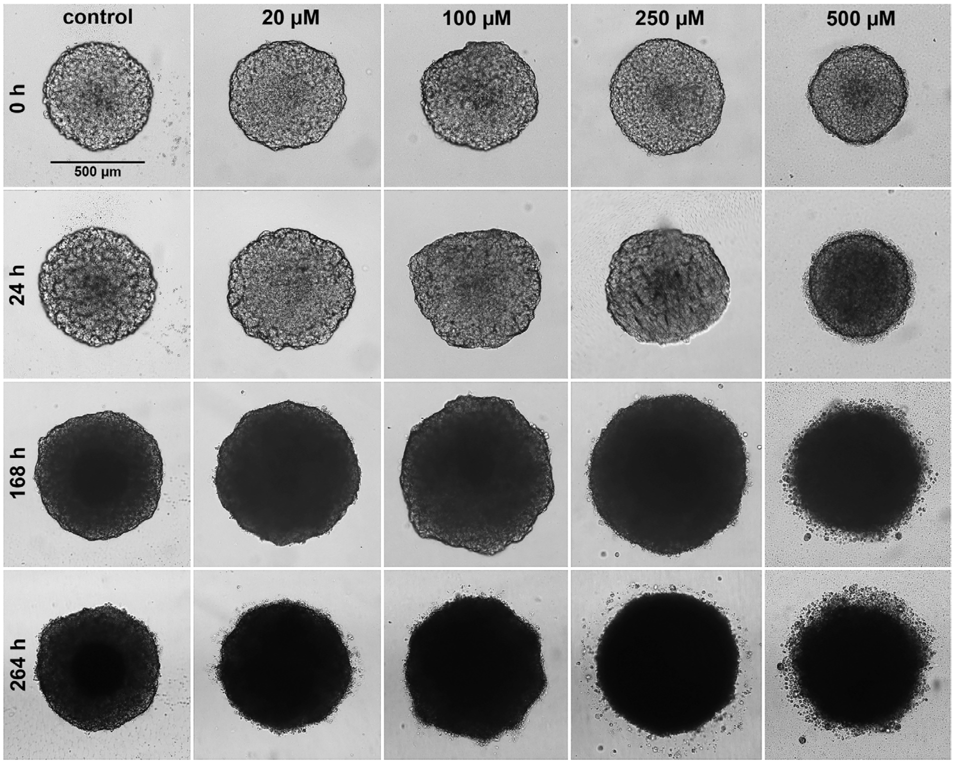

Supplementary Figure 12: Effect of palmitoyl-lysophosphatidylcholine LPC[16:0] on tumor spheroid invasion and growth. 3D cell spheroids of hypopharynx squamous cell carcinoma origin were embedded in Matrigel matrix. Invasion and growth of spheroids are shown in the presence of increasing concentration of LPC[16:0] at different time points after embedding. Example images of spheroid cross-sections are shown.

Supplementary Table 1: Proteomic parameters of the identified proteins from the microbiopsy of HNSCC neoplastic region

Accesion No.	Protein name	MW/kDa	Mascot score	Peptides	SC %
gi|83754467	Chain A, Molecular Basis For The Recognition Of Phosphorylated And Phosphoacetylated Histone H3	29.24	118	24	43.41
gi|51859376	H3 histone, family 3A	15.27	116	19	51.47
gi|18645167	Annexin A2, isoform 2	38.55	116	25	52.51
gi|119597993	annexin A2, isoform CRA_c	32.42	91	17	43.55
gi|3660145	Chain B, Crystal Structure Of S-Nitroso-Nitrosyl Human Hemoglobin A	15.86	90	10	75.34
gi|300508775	Chain B, Human Hemoglobin A Mutant Beta H63w Carbonmonoxy-Form	15.90	90	11	84.25
gi|194374253	unnamed protein product	14.04	90	16	45.53
gi|358440049	Chain A, Crystal Structure Of Human Galectin-7 In Complex With A Galactose-Benzylphosphate Inhibitor	14.73	90	9	45.11
gi|119610321	hCG1749005	16.17	90	15	44.37
gi|4757756	annexin A2 isoform 2	38.57	89	19	38.64
gi|347447337	Chain B, Crystal Structure Of Human Nucleosome Core Particle Containing H4k44q Mutation	11.64	87	17	87.74
gi|662841	heat shock protein 27	22.31	87	12	46.73
gi|4504517	heat shock protein beta-1	22.76	87	13	48.78
gi|4504299	histone H3.1t	15.49	87	15	47.79
gi|119618340	hypothetical protein MGC15619, isoform CRA_b	36.85	85	17	23.13
gi|1166436	histone H3.3	6.89	85	12	63.33
gi|30354619	YWHAZ protein, partial	35.31	81	25	36.88
gi|124504316	HIST2H4B protein	11.37	80	16	81.37
gi|56203471	histone cluster 2, H3, pseudogene 2	15.42	80	16	46.32
gi|119575948	histone 1, H4e	15.28	80	18	69.57
gi|229361	Myoglobin	17.04	79	12	63.40
gi|414587	ribosomal protein L10	23.90	78	17	47.80
gi|61679604	Chain B, T-To-T (High) Quaternary Transitions In Human Hemoglobin: Deshis146beta Deoxy Low-Salt	15.72	78	11	84.14
gi|46014946	Chain B, Crystal Structure Of Human Hemoglobin E At 1.73 A Resolution	15.85	78	11	78.08
gi|12006350	60S ribosomal protein L15	24.14	77	17	47.55
gi|296863397	Chain B, The Nucleosome Containing A Testis-Specific Histone Variant, Human H3t	11.64	77	17	84.91
gi|480312323	Chain L, Crystal Structure Of Broadly And Potently Neutralizing Antibody 3bnc117 In Complex With Hiv-1 Gp120	22.98	77	10	37.86
gi|358440049	Chain A, Crystal Structure Of Human Galectin-7 In Complex With A Galactose-Benzylphosphate Inhibitor	14.73	76	8	55.64
gi|45219796	Histone cluster 1, H3i	15.41	75	14	45.59
gi|4504517	heat shock protein beta-1	22.76	75	11	40.98
gi|999565	Chain B, Oxygen Affinity Modulation By The N-Termini Of The Beta Chains In Human And Bovine Hemoglobin	15.75	71	9	62.07
gi|307776567	Chain A, Structure Of 14-3-3 Isoform Sigma In Complex With A C-Raf1 Peptide And A Stabilizing Small Molecule Fragment	26.75	71	20	40.17
gi|3660145	Chain B, Crystal Structure Of S-Nitroso-Nitrosyl Human Hemoglobin A	15.86	71	9	62.33
gi|453056145	Chain A, Molecular Tweezers Modulate 14-3-3 Protein-protein Interactions	26.49	70	21	44.07
gi|410170435	uncharacterized protein LOC101060017	34.27	70	21	35.17
gi|194390512	unnamed protein product	17.82	70	12	56.33
gi|51094756	similar to t-complex 1; T-complex locus TCP-1; t-complex 1 (a murine tcp homolog)	19.81	70	12	48.35
gi|3660145	Chain B, Crystal Structure Of S-Nitroso-Nitrosyl Human Hemoglobin A	15.86	69	9	62.33
gi|194374253	unnamed protein product [Homo sapiens]	14.04	68	11	45.53
gi|71042776	Chain A, 14-3-3 Protein Theta (Human) Complexed To Peptide	29.17	67	18	31.25
gi|410171656	PREDICTED: uncharacterized protein LOC101060667	39.14	67	22	34.06
gi|218783334	immunoglobulin light chain	23.15	65	10	46.26
gi|148664230	ankyrin repeat and LEM domain-containing protein 2	104.04	65	24	16.95
gi|99031801	Chain L, Crystal Structure Of A Glycosylated Fab From An Igm Cryoglobulin With Properties Of A Natural Proteolytic Antibody	23.33	65	10	46.05
gi|11275302	anti TNF-alpha antibody light-chain Fab fragment	23.50	65	9	40.65
gi|258588258	Chain A, Germline V-Genes Sculpt The Binding Site Of A Family Of Antibodies Neutralizing Human Cytomegalovirus	23.68	65	10	45.83
gi|21614544	protein S100-A8	10.82	65	7	50.54
gi|62897717	lactate dehydrogenase A variant	36.66	64	12	32.23
gi|1166436	histone H3.3	6.89	63	7	46.67
gi|42476296	tropomyosin 2 (beta) isoform 1	32.83	63	16	42.61
gi|119618339	hypothetical protein MGC15619, isoform CRA_a	37.31	63	15	20.74
gi|155030216	sister chromatid cohesion protein PDS5 homolog A isoform 1	150.73	62	29	13.01
gi|1066082	DNA-binding protein	25.78	60	11	34.84
gi|119602933	hCG18094	79.40	60	24	20.35

Supplementary Table 2: Data and patient info for samples used in this study. See Supplementary Table 2
